# Evaluation of Four Adjuvant Combinations, IVAX-1, IVAX-2, CpG-1826+Montanide ISA 720 VG and CpG-1018+Montanide ISA 720 VG, for Safety and for Their Ability to Elicit Protective Immune Responses in Mice against a Respiratory Challenge with *Chlamydia muridarum*

**DOI:** 10.3390/pathogens12070863

**Published:** 2023-06-22

**Authors:** Sukumar Pal, Anatoli Slepenkin, Jiin Felgner, D. Huw Davies, Philip Felgner, Luis M. de la Maza

**Affiliations:** 1 Department of Pathology and Laboratory Medicine, University of California, Irvine, CA 92697, USA; spal@uci.edu (S.P.); aslepenk@uci.edu (A.S.); 2 Vaccine Research and Development Center, Department of Physiology and Biophysics, University of California, Irvine, CA 92697, USA; felgnerj@hs.uci.edu (J.F.); ddavies@uci.edu (D.H.D.); pfelgner@hs.uci.edu (P.F.)

**Keywords:** *Chlamydia trachomatis*, *Chlamydia muridarum*, vaccine, adjuvant combinations, major outer membrane protein, mice

## Abstract

There is an urgent need to produce a vaccine for *Chlamydia trachomatis* infections. Here, using the *Chlamydia muridarum* major outer membrane protein (MOMP) as an antigen, four adjuvant combinations IVAX-1 (MPLA+CpG-1018+AddaVax), IVAX-2 (MPLA+CpG-1018+AS03), CpG-1826+Montanide ISA 720 VG (CpG-1826+Mont) and CpG-1018+Montanide ISA 720 VG (CpG-1018+Mont), were tested for their local reactogenicity and ability to elicit protection in BALB/c mice against a respiratory challenge with *C. muridarum.* Immunization with IVAX-1 or IVAX-2 induced no significant local reactogenicity following intramuscular immunization. In contrast, vaccines containing Montanide resulted in the formation of a local granuloma. Based on the IgG2a/IgG1 ratio in serum, the four adjuvant combinations elicited Th1-biased responses. IVAX-1 induced the highest in vitro neutralization titers while CpG-1018+Mont stimulated the lowest. As determined by the levels of IFN-γ produced by T-cells, the most robust cellular immune responses were elicited in mice immunized with CpG-1018+Mont, while the weakest responses were mounted by mice receiving IVAX-1. Following the respiratory challenge, mice immunized with CpG-1018+Mont lost the least amount of body weight and had the lowest number of *C. muridarum* inclusion-forming units (IFUs) in the lungs, while those receiving IVAX-2 had lost the most weight and had the highest number of IFUs in their lungs. Animals vaccinated with CpG-1826+Mont had the lightest lungs while those immunized using IVAX-2 had the heaviest. To conclude, due to their safety and adjuvanticity, IVAX formulations should be considered for inclusion in human vaccines against Chlamydia.

## 1. Introduction

The World Health Organization has estimated that 130 million individuals worldwide are infected in the genitourinary tract each year with *Chlamydia trachomatis,* making it the most common sexually transmitted bacterial pathogen [1,2]. In addition, *C. trachomatis* causes ocular, respiratory, and gastrointestinal infections [3,4].

To protect against *C. trachomatis*-induced trachoma, whole-pathogen inactivated vaccines were tested in several countries [3,5]. Some vaccine formulations protected against trachoma. Protection, however, was found to be short-lived (1–3 years) and serovar/serogroup-specific. In addition, some vaccinees developed a hypersensitivity reaction upon re-exposure to this pathogen [3,5,6,7,8]. Although the cause of this hypersensitivity reaction is still under investigation, the possibility that an antigenic component of *Chlamydia* mediated this adverse outcome stimulated the search for a subunit vaccine [2,9,10,11,12,13].

DNA sequencing of *C. trachomatis* identified the major outer membrane protein (MOMP) as the likely antigen that induced serovar/serogroup-specific protection during the trachoma vaccine trials [14]. Since then, a number of chlamydial antigens have been tested for their ability to confer protection against genital, respiratory and ocular challenges [10,12,13,15,16]. Of these, MOMP is still the most promising candidate [9,17,18,19,20,21,22,23,24]. MOMP is highly antigenic containing multiple B-cell epitopes, mostly located in the variable domains, while the T-cell epitopes are for the most part in the constant domains [14,25,26]. The amino acid sequence of the variable domains (VDs) defines each serovar [14,26,27].

In contrast to whole-cell vaccines, which are self-adjuvanted, a shortcoming of subunit vaccines is that they lack the adjuvant activity necessary to induce innate and adaptive immune responses following immunization [28]. In mice, both humoral and cell-mediated immune responses contribute to protection against *Chlamydia* [29,30,31]. Thus, there is a need to identify the adjuvants, or their combinations, that elicit mucosal and systemic humoral and cellular immune responses. Safety is a major concern for vaccines and therefore, adjuvants need to be tested in animal models to make sure that they do not produce significant local or systemic toxicity or reactogenicity. Here, to achieve these goals, we tested four adjuvant combinations.

To compare the safety and effectiveness of the adjuvant combinations, CpG-1826 and CpG-1018 were tested with Montanide ISA 720 VG. The CpG adjuvants are cytosine phosphoguanine motifs that mimic bacterial and viral nucleic acids. CpG-1826 and CpG-1018 are agonists of TLR-9 and induce Th1-biased immune responses with direct activation of monocytes, macrophages and dendritic cells (DCs) that secrete IL-6, IL-12, IFN-γ, TNF-α and several chemokines [32,33]. Furthermore, CpG stimulates B-cells to proliferate and secrete immunoglobulins, IL-6 and IL-12. The overall effect of CpG is the induction of strong Th1 humoral and cellular immune responses and the broadening of B-cell epitope recognition. CpG-1826 has been optimized to induce robust immune responses in mice but not in humans. CpG-1018 plus Montanide ISA 720 (CpG-1018+Mont) has been found to be a very effective combination to elicit protective immune responses in mice against a chlamydial challenge [22,34,35]. CpG-1018, unlike CpG-1826, elicits a robust immune response in both humans and mice and has recently been approved by the FDA for use in a hepatitis B virus vaccine [36]. Therefore, here we tested CpG-1018 in parallel with CpG-1826, both in combination with Montanide ISA 720 VG, to compare their safety and efficacy.

Montanide ISA 720 VG is a non-TLR adjuvant containing squalene that promotes the recruitment of antigen-presenting cells (APCs) and the phagocytic uptake of the antigen [37]. This results in increases in antibody responses. Montanide ISA 720 VG, when formulated as “a water-in-oil” suspension, produces reactogenicity at the site of immunization. In spite of this limitation, it has been used in Phase I and II clinical trials with vaccine candidates for HIV-1, SARS-CoV-2, malaria and cancer [38,39,40,41,42].

Hernandez-Davies et al. [43] recently tested in C57BL/6 mice nine different TLR agonist combinations, with or without AddaVax, using the influenza A hemagglutinin trimer as the antigen. Among the 18 new adjuvant combinations, the formulation containing CpG-1018, monophosphoryl-Lipid A (MPLA) and AddaVax (termed IVAX-1) was found to elicit the most robust immune responses. MPLA is a TLR-4 agonist that signals via the TRIF adaptor and induces preferentially Th-1-biased responses [44,45,46]. It is a less toxic derivative of the lipid A from *Salmonella minnesota* R595 lipopolysaccharide. By enhancing APC maturation, MPLA improves the immunogenicity of vaccine antigens. MPLA is part of the ASO4 adjuvant used in licensed human vaccines (Fendrix, and Cervarix). AddaVax is a squalene-based oil-in-water emulsion with a formulation similar to that of MF59, which is an adjuvant approved for use in influenza vaccines [47]. AddaVax stimulates the recruitment and activation of APCs and the production of cytokines and chemokines by granulocytes and macrophages, which increase antibody responses [42,43].

IVAX-1, as determined by the ratio of IgG2c/IgG1 and the production of IFN-γ by CD4 and CD8 T-cells, was the most effective at eliciting Th1-biased responses [43]. This adjuvant combination increased the titers and the breadth of antibodies to hemagglutinin and the level of in vitro neutralizing antibodies. Importantly, no significant local or systemic reactogenic effects were noted, and similar immune responses were obtained when IVAX-1 was delivered by subcutaneous and intranasal routes.

IVAX-2 is a new adjuvant combination that, like IVAX-1, was formulated with the goal of eliciting strong humoral immune responses with influenza A hemagglutinin [43]. It is similar to IVAX-1 but contains ASO3 instead of AddaVax. ASO3 is an oil-in-water emulsion containing squalene, DL-α-tocopherol (Vitamin E) and polysorbate-80 and is approved for human use [46,48]. AS03 triggers NF-κB-dependent innate immune responses releasing cytokines and chemokines at the site of injection and in the draining lymph nodes. Monocytes and granulocytes then migrate, leading to increases in antigen-specific antibody responses.

Here, we used the murine challenge respiratory model to identify the formulations of Montanide ISA 720 VG + CpG-1018, Montanide ISA 720 VG + CpG-1826, IVAX-1 and IVAX-2 that were safe and induced the most robust protective immune responses. The goal was to find a vaccine formulation that can be tested in the mouse genital challenge model and eventually in humans. In comparison with the respiratory model, testing chlamydial vaccines in the mouse genital tract infection model is lengthy, time-consuming, and the personnel and supplies are expensive. The reactogenicity/toxicity/immunology of the vaccine formulation can equally be tested in the respiratory and genital tract models. Here, we used the i.m. route for immunization, because this will likely be the route used to deliver a vaccine to protect against chlamydial genital infections and we can test the possible negative effects at the site of injection. The respiratory and the genital tract, although quite different anatomically and physiologically, have similarities, including a mucosal and a systemic component that are affected during infection by *C. trachomatis.* Therefore, this study allowed us to evaluate the safety and the protective immune responses in both compartments. Furthermore, in humans, *C. trachomatis* infections can occur when a newborn is infected in the birth canal and develops pneumonia a few weeks later [49]. *C. trachomatis* respiratory infections have also been described in adults, particularly in those who are immunocompromised [50,51]. In addition, *Chlamydia pneumoniae* and *Chlamydia psittaci* are common respiratory pathogens in humans, and information learned from testing *C. trachomatis* vaccines in the mouse respiratory model will likely help to formulate vaccines against these pathogens [52,53,54,55,56,57,58].

In this study, with the goal of identifying a vaccine formulation that was safe and elicited robust humoral and cell-mediated protective immune responses against a respiratory challenge with *C. muridarum*, MOMP was delivered with four adjuvant combinations. Three of them, IVAX-1, IVAX-2 and CpG-1018+Mont, have never been tested before in vaccines to protect against *Chlamydia* infections. We hypothesized that the adjuvant combination that elicits the most robust Th1-biased immune responses will be the most protective.

## 2. Materials and Methods

### 2.1. Stocks of C. muridarum

*C. muridarum* (strain NiggII; ATCC) was grown in HeLa-229 cells using high-glucose Dulbecco’s medium, plus cycloheximide (1 µg/mL) and gentamycin (10 µg/mL), without fetal bovine serum. Elementary bodies (EBs) were purified and stored in sugar phosphate glutamate buffer (SPG) at −80 °C [59]. The number of *C. muridarum* inclusion-forming units (IFUs) was determined in HeLa-229 cells as described in [17].

### 2.2. Cloning, Expression and Purification of C. muridarum MOMP

The method to clone, express and purify *C. muridarum* MOMP was published in [18]. Briefly, the *C. muridarum* MOMP gene (GenBank, accession No. AE002272, X63409), without the leader sequence, was amplified by PCR and inserted into the pET-45b(+) vector (Novagen, Madison, WI, USA). *Escherichia coli* TOP10 competent cells were transformed, and the plasmid was extracted from positive clones. For expression, *E. coli* BL21 (DE3) was transformed with the plasmid containing *C. muridarum* MOMP DNA and inoculated into LB broth. MOMP was extracted from *E. coli* inclusion bodies and purified using a Sephacryl-S-300 column (Sigma-Aldrich, St. Louis, MO) column (1 × 50 cm) [16]. By the Limulus amoebocyte assay (Associates of Cape Cod Inc.; East Falmouth, MA, USA), MOMP had less than 0.05 EU of LPS/mg of protein.

### 2.3. Mice Vaccination, Intranasal Challenge and Assessment of the Course of the Disease and the C. muridarum Infection in Mice

Four-to-five-week-old female BALB/c (H-2^d^) mice (Charles River Laboratories, Wilmington, MA, USA) were immunized with *C. muridarum* MOMP (10 μg/mouse/immunization), twice at four-week intervals by the intramuscular (i.m.) route in the quadriceps muscle (Figure S1 and Table S1). The following adjuvant combinations were used with MOMP: (1) CpG-1826 (10 µg/mouse/immunization) (Tri-Link) + Montanide ISA 720 VG (70:30 *v/v*) (Seppic, Inc., Fairfield, NJ, USA); (2) CpG-1018 (10 µg/mouse/immunization) (Integrated DNA Technologies; Coralville, IA, USA) + Montanide ISA 720 VG (70:30 *v/v*); (3) IVAX-1: MPLA (5.3 μg/mouse/immunization, i.e., 3 nmole standard dose formulated as liposome along with DOPG co-lipid) (Avanti Polar Lipids Inc.; Alabaster, AL, USA) + CpG-1018 (7.15 μg/mouse/immunization, i.e., 1 nmole standard dose (Integrated DNA Technologies) + AddaVax (50 μl/mouse/immunization) (Invivogen Inc., San Diego, CA, USA)) and (4) IVAX-2: (MPLA (5.3 μg/mouse/immunization, i.e., 3 nmole standard dose formulated as liposome along with DOPG co-lipid) + CpG-1018 (7.15 μg/mouse/immunization, i.e., 1 nmole standard dose, (Integrated DNA Technologies) + AS03 50 μl/mouse/immunization) (Invivogen Inc., San Diego, CA, USA). The two IVAX formulations were recently developed and tested in Drs. Felgner and Davies labs [43] (Supplemental Figure S1 and Supplemental Table S1). A negative immunization control group received PBS only, and an adjuvant negative control was vaccinated only with MOMP (10 μg/mouse/immunization). The positive antigen control received i.n. 10^4^ *C. muridarum* IFUs once at the time of the first immunization. A total of 12–13 mice per group were immunized. Three mice/group were used to characterize the cell-mediated immune responses before the challenge and 9–10 mice were challenged intranasally with *C. muridarum*. The local reactogenicity at the site of immunization was visually evaluated when the mice were euthanized, and the size of the local reaction was measured.

Four weeks after the second immunization, anesthetized mice were challenged i.n. with 10^4^ IFUs of *C. muridarum* [60]. Animals were weighed daily for 10 days post-challenge (d.p.c.) when they were euthanized and their lungs weighed and homogenized (Seward Stomacher 80; Seward, Laboratory System Inc., Bohemia, NY, USA) in 5 mL of SPG/mouse. To determine the number of *C. muridarum* IFUs present in the lungs, six 10-fold serial dilutions of the lung homogenates were used to infect Hela-229 cells grown in 48-well plates. Following incubation for 30 h at 37 °C in a CO_2_ incubator, the chlamydial IFUs were visualized with mAb MoPn-40 and counted using a light microscope [61]. The limit of detection (LD) was <50 *C. muridarum* IFU/lungs/mouse.

To evaluate the local cellular immune responses, levels of IFN-γ in lung supernatants collected from lung homogenates at 10 d.p.c. were determined by ELISA as described [62]. To assess the humoral immune responses in the lungs at 10 d.p.c., the levels of *C. muridarum*-specific IgA were determined in the lung supernatants [63].

The University of California, Irvine, IACUC (AUP-20-054) approved the vertebrate animal protocols.

### 2.4. Determination of the Humoral Immune Responses following Vaccination

Blood from the periorbital plexus was collected before vaccination and the day before the respiratory challenge. Antibody titers to *C. muridarum* EBs (1 μg/well) were determined by ELISA using 96-well plates as described in [64]. Serial dilutions of serum were added, and the antigen–antibody reactions were detected with HRP-conjugated goat anti-mouse. Goat anti-mouse IgG, IgG1, and IgG2a (BD Bioscience, San Diego, CA, USA) diluted 1:5000 for IgG and 1:1000 for the two isotypes were used. ABTS [2,2′-azino-bis-(3-ethylbenzthiazoline-6-sulfonate)] (Sigma-Aldrich, St. Louis, MO, USA) was utilized as the substrate and the plates were scanned in an ELISA reader at 405 nm (Labsystem Multiscan; Helsinki, Finland). Titers were calculated using the OD of pre-immunization sera ± 2 SD as a background and reported as geometric mean titer (GMT).

In vitro neutralization assays were performed as previously described [65]. Briefly, duplicate sets of two-fold serial dilutions of serum were made in Ca^+2^/Mg^+2^-free PBS containing 5% guinea pig serum as a source of complement. Serum samples were incubated with 10^4^ *C. muridarum* IFUs for 45 min at 37 °C and centrifuged onto HeLa-229 monolayers grown in flat-bottom 96-well plates. Monolayers were incubated for 30 h in culture medium with cycloheximide (1 µg/mL). Cells were fixed with methanol and chlamydial IFUs were stained with mAb MoPn-40 [66]. The number of IFUs was counted and neutralization was defined as greater than or equal to a 50% decrease in the number of IFUs when compared with the controls incubated with pre-immunization sera.

To examine the presence of antibodies to linear epitopes following vaccination, overlapping 25-mers, corresponding to the mature *C. muridarum* MOMP amino acid sequence, were chemically synthesized (SynBioSci Corp.; Livermore, CA, USA). Peptide 25 (p25) overlaps with the *N*- and *C*-terminus of MOMP. The peptides were adsorbed onto high-binding-affinity ELISA plates (1 µg/well of a 96-well plate) and the antibody binding was determined in triplicate using anti-mouse IgG as described in [67].

To evaluate the humoral immune responses to *C. muridarum* EBs in the genital mucosa, vaginal washes were collected the day before the i.n. challenge and levels of IgG and IgA were determined as discussed above. Two pools of vaginal washes, five mice each, were run in triplicate.

### 2.5. Evaluation of Memory-Cell-Mediated Immune Responses following Vaccination

The day before the respiratory challenge, splenic T-cells, purified using nylon wool (85–90% purity), were stimulated with *C. muridarum* EBs in the presence of antigen-presenting cells (APCs) [61]. APCs were prepared by the irradiation (3300 rads, ^137^Cs) of syngeneic splenocytes, and 2.5 × 10^6^ cells were incubated in flat-bottom 48-well plates (Costar, Corning Inc., NY, USA) at 37 °C for 2 h with EBs at a 1:1 ratio. T-cells were added to APCs at a ratio of 1:1. Concanavalin A (5 μg/mL) served as a positive stimulant and cell culture medium (RPMI with 10% FBS) was used as a negative antigen control. Levels of IFN-γ and IL-4 in supernatants from EB-stimulated T-cells were determined using commercial kits (BD Pharmingen, San Diego, CA, USA) [64,68].

### 2.6. Statistical Analyses

The Mann–Whitney *U* test was used to compare antibody titers in serum, the number of *C. muridarum* IFUs in the lungs, and levels of IFN-γ in lung supernatants. Repeated-measures ANOVA was employed to compare changes in mean body weight over 10 days following the *C. muridarum* i.n. challenge. The Student’s *t*-test was utilized to evaluate differences between changes in body weight at 10 d. p.c., lung weights, levels of IFN-γ and IL-4 in T-cell recall assays, and levels of IgA in lung supernatants. A *p*-value of < 0.05 was considered to be significant. A *p*-value of <0.10 was deemed as approaching significance.

## 3. Results

### 3.1. Assessment of the Immune Reaction at the Site of Vaccination

**Figure 1:**
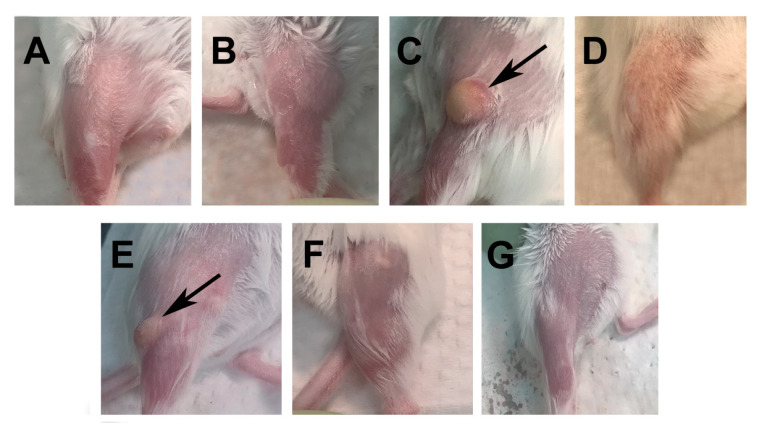
Evaluation of the reactogenicity/toxicity at the site of vaccination. Before the mice were euthanized, pictures were taken of the site where the vaccines were delivered. (**A**) No injection control; (**B**) PBS control; (**C**) CpG-1826+Mont/MOMP; (**D**) IVAX-1/MOMP; (**E**) CpG-1018+Mont/MOMP; (**F**) IVAX-2/MOMP; (**G**) MOMP only.

To evaluate the local reactogenicity, pictures were taken on the last day of the experiment when the mice were euthanized. Figure 1A,B shows the controls not immunized and receiving PBS, respectively. As shown in Figure 1C,E, the mice immunized with CpG-1826+Mont or with CpG-1018+Mont developed granulomas measuring approximately 2–5 mm in size. In contrast, the mice immunized with IVAX-1 or IVAX-2 (Figure 1D,F) showed no abnormalities at the site of immunization. The mice only receiving MOMP had no local reaction (Figure 1G). No significant negative effects were observed in the physical activity or behavior of the mice receiving any of the four vaccine formulations.

### 3.2. Humoral Immune Responses Elicited by Vaccination

**Figure 2:**
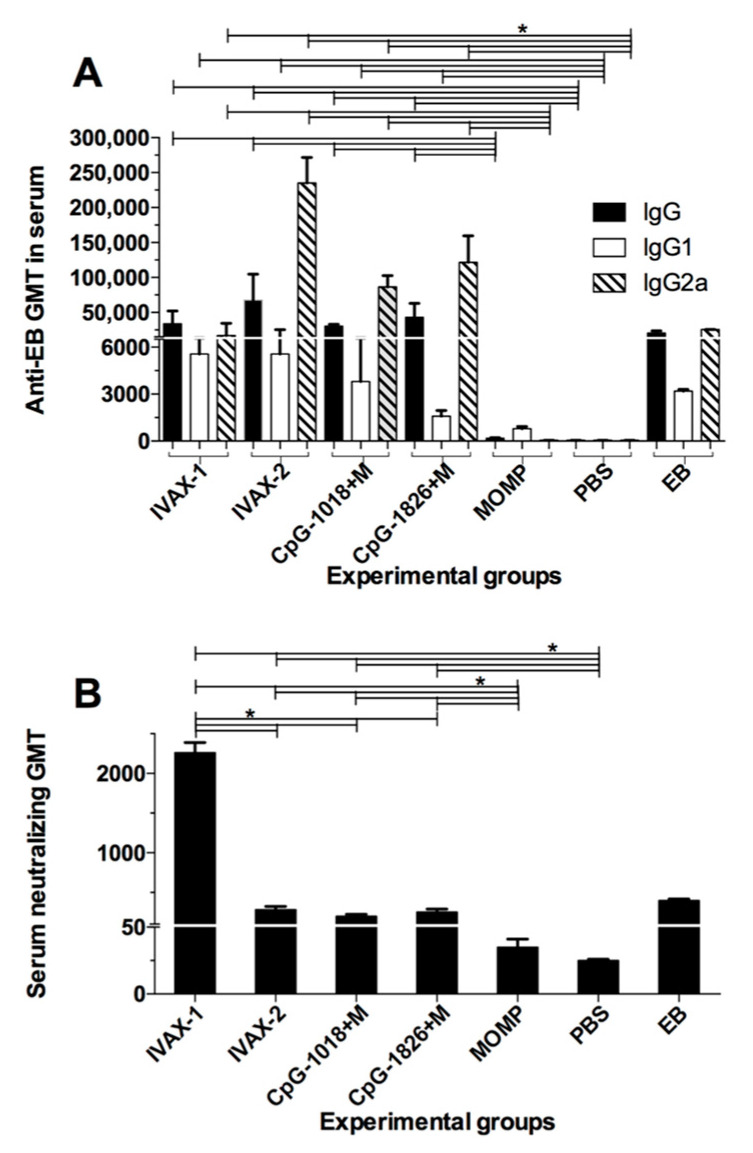
Antibody responses following immunization. (**A**) IgG, IgG1 and IgG2a ELISA titers (GMT ± SE) to *C. muridarum* EBs. Mice were immunized and blood was collected the day before the i.n. challenge. (**B**) In vitro neutralizing antibody GMT ± SE using serum collected before the challenge. Pre-immunization sera were used as negative controls. * *p* < 0.05 by the Mann–Whitney *U* test.

To determine the vaccine-induced humoral immune responses, serum samples were collected from mice the day before the i.n. challenge. Pre-immunization sera were used as controls. Antibody geometric mean titers (GMTs) were determined using EBs as the antigen (Figure 2A). The positive antigen control immunized i.n. with viable *C. muridarum* EBs had an IgG GMT of 25,600, while in the negative control receiving PBS the GMT was below the limit of detection (<100). The mice vaccinated with MOMP, without adjuvants, had a GMT of 1600, while the mice immunized with MOMP and one of the four adjuvant combinations had GMTs that ranged from 229,880 to 540,470. No significant differences in titer were determined between these four groups of mice.

To establish whether the vaccines elicited Th1- or Th2-biased immune responses, IgG2a and IgG1 antibodies were quantified, respectively (Figure 2A). The mice immunized with *C. muridarum* EBs had a ratio of 16 (25,600/1600), while those vaccinated only with MOMP had ratio of <0.08 (<100/1270). Of the experimental groups, the one immunized using CpG-1018+Mont had the highest IgG2a/IgG1 ratio of 80, while a more balanced Th1/Th2 response, with a ratio of 5, was observed in the mice receiving IVAX-1.

The in vitro neutralizing antibody GMT in the serum was determined the day before the challenge (Figure 2B). Serum collected before immunization was used as the negative control. The in vitro neutralization GMT in mice immunized with EBs was 400, while for the MOMP only and PBS-vaccinated groups, it was below the level of detection (<50). The highest in vitro neutralization GMT was observed in the mice vaccinated with IVAX-1 (2263). The other three experimental groups had GMTs ranging from 200 to 283, significantly lower than the titer elicited by IVAX-1 (*p* < 0.05).

**Figure 3:**
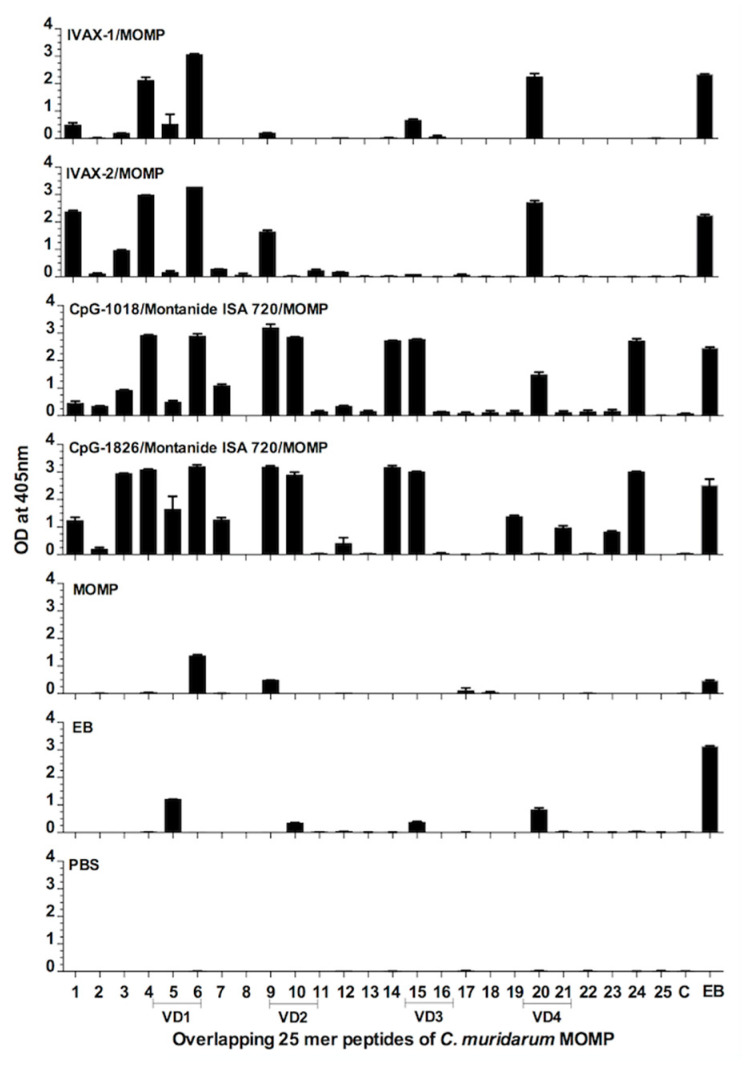
Binding of serum antibodies from immunized mice to synthetic *C. muridarum* MOMP peptides. Serum samples from mice were collected the day before the i.n. challenge. Their reactivity to 25-aa overlapping peptides corresponding to the *C. muridarum* mature MOMP was analyzed by ELISA.

IgG antibodies to the four *C. muridarum* MOMP variable domains (VDs) were identified in serum from the positive control immunized with EBs and the groups vaccinated with CpG-1018+Mont or CpG-1018+Mont (Figure 3). Overall, the mice vaccinated with IVAX-1 or IVAX-2 generated weak immune responses against the MOMP VD. This was particularly evident when evaluating the antibody responses to VD2 and VD3. MOMP, without adjuvants, did not elicit responses to VD3 or VD4.

**Figure 4:**
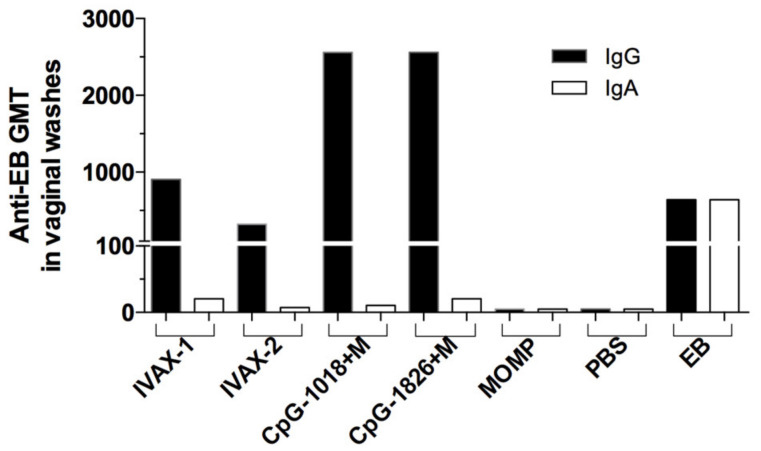
IgG and IgA antibody titers in pooled vaginal washes collected the day before the i.n. challenge. Vaginal washes were collected, two pools of five mice/group were run in triplicate, and the levels of IgG and IgA were determined using *C. muridarum* EBs.

To determine mucosal IgG and IgA antibody titers to EBs, vaginal washes were collected and pooled (Figure 4). For the four groups immunized with MOMP, the IgG titers were low and the IgA titers were below the level of detection. EB-immunized mice had similar levels of IgG and IgA.

### 3.3. Memory Cellular Immune Responses Induced by Vaccination

**Figure 5:**
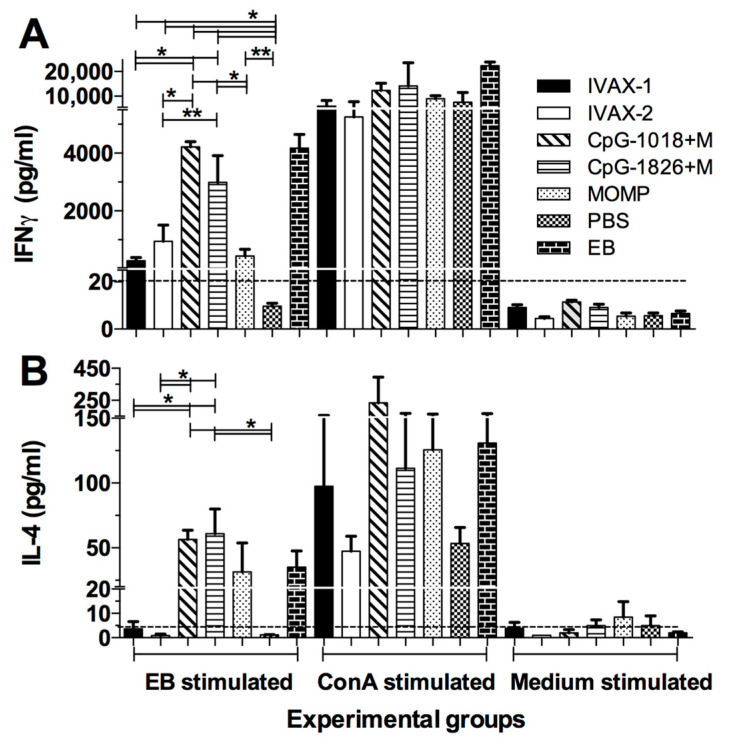
Determination of (**A**) IFN-γ and (**B**) IL-4 levels in T-cell supernatants collected from vaccinated mice the day before the i.n. challenge. The day before the intranasal challenge, four randomly selected mice from each group were euthanized, their spleens collected, and T-cells isolated using nylon wool columns and stimulated with *C. muridarum* EBs, with Concanavalin A as a non-specific stimulant, or with medium as a negative control. * *p* < 0.05 and ** *p* < 0.1 by the Student’s *t*-test. The limit of detection is indicated with a broken horizontal line.

As a parameter of the *C. muridarum*-specific memory cellular immune responses elicited by the vaccines, purified spleen T-cells were stimulated with EBs and the levels of cytokines were determined in the supernatants (Figure 5A,B). T-cells from the control animals immunized with *C. muridarum* EBs secreted high levels of IFN-γ (4179 ± 480 pg/mL) compared with the negative control mice receiving PBS (<15 pg/mL) or MOMP only (431 ± 232) (*p* < 0.05). Of the groups immunized with adjuvant combinations, the mice vaccinated with CpG-1018+Mont produced the highest levels of IFN-γ (4213 ± 183 pg/mL), followed by the group immunized with CpG-1826+Mont (2989 ± 925 pg/mL). The mice vaccinated with IVAX-1 (271 ± 114 pg/mL) or IVAX-2 (935 ± 561 pg/mL) had significantly lower levels of IFN-γ than those immunized with CpG-1018+Mont (*p* < 0.05). Indicative of cellular viability, all groups stimulated with ConA produced high levels of IFN-γ, while the medium-stimulated T-cells did not produce IFN-γ (limit of detection 20 pg/mL).

Low levels of IL-4 were detected in the T-cell supernatants from mice vaccinated with CpG-1018+Mont or with CpG-1018+Mont. No IL-4 was found in the supernatants from mice immunized with IVAX-1 or IVAX-2 (limit of detection 4 pg/mL). The T-cells stimulated with ConA produced IL-4, while those stimulated with medium did not.

### 3.4. Changes in Body Weight of Mice following the C. muridarum i.n. Challenge

**Figure 6:**
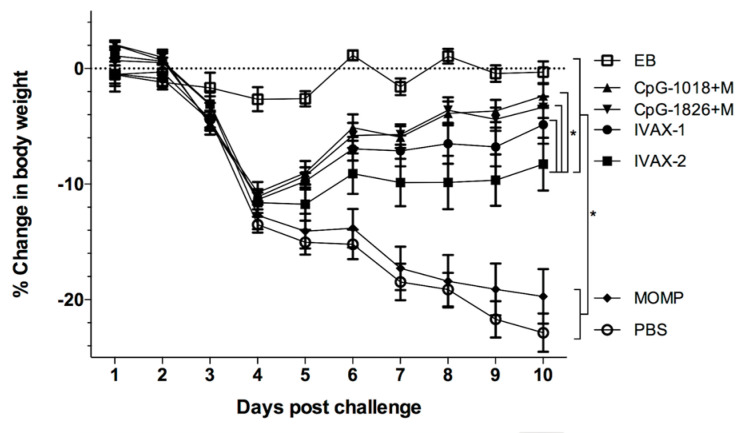
Daily changes in mean body weight following the i.n. challenge with 10^4^ *C. muridarum* IFUs. Percentage changes in daily mean body weight following the i.n. challenge with *C*. *muridarum.* * *p* < 0.05 by the repeated-measures ANOVA.

As a measurement of the systemic effect, the body weight was determined for 10 days following the i.n. challenge with 10^4^ inclusion-forming units (IFUs) of *C. muridarum*. Except for the positive control group immunized i.n. with live EBs, all the mice lost weight from day two to four p.c. (Figure 6). Subsequently, in contrast to the mice immunized with MOMP only or PBS, the animals vaccinated with adjuvanted *C. muridarum* MOMP slowly regained most of their initial body weight. As determined by the repeated-measures ANOVA, the cumulative body weight changes over the 10 days were significantly different between the mice immunized with *C. muridarum* MOMP and adjuvant combinations and the two negative control groups receiving MOMP only or PBS (*p* < 0.05). The mice immunized with CpG-1018+Mont, CpG-1018+Mont, or IVAX-1 lost less body weight over the 10-day period than the groups immunized with IVAX-2 (*p* < 0.05).

**Figure 7:**
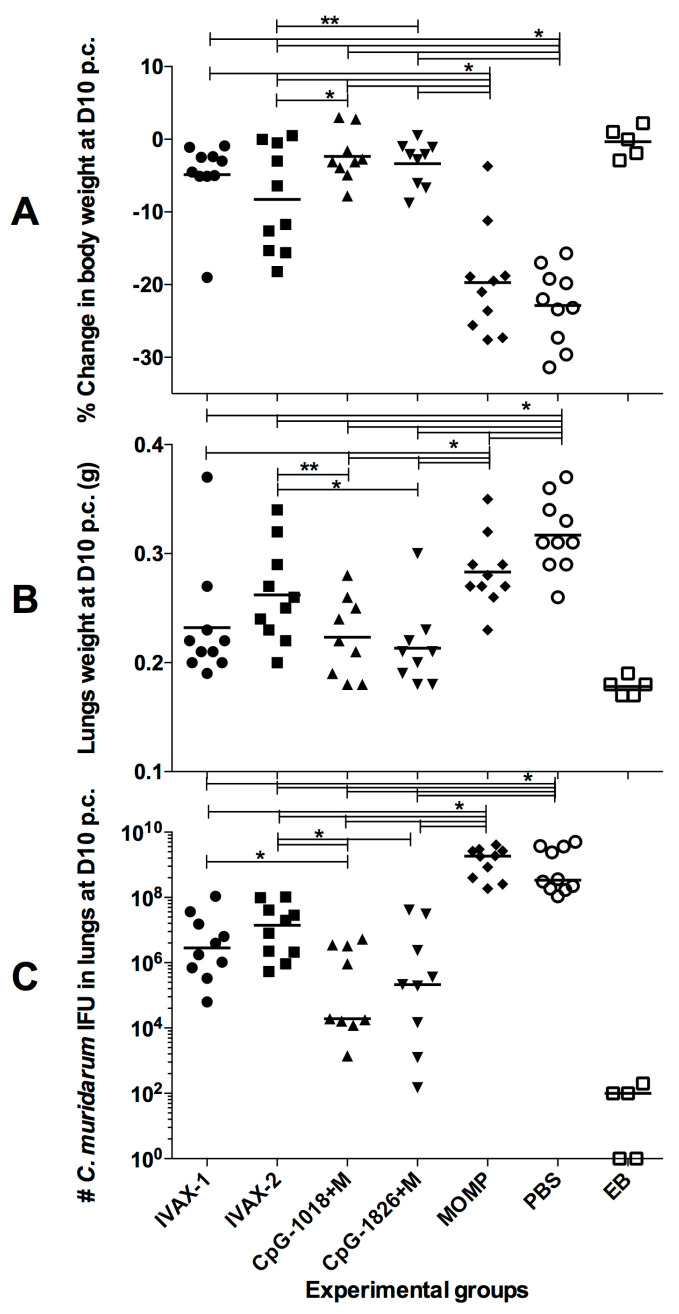
Disease burden at day 10 following the i.n. challenge with 10^4^ *C. muridarum* IFUs. (**A**) Percentage change in body weight at 10 days following the i.n. challenge. The mean is shown as a horizontal line. Each symbol represents an animal. * *p* < 0.05 and ** *p* < 0.1 by the Student’s *t*-test. (**B**) Lung weights (g) at 10 days after the i.n. challenge. The mean is shown as a horizontal line. Each symbol represents an animal. * *p* < 0.05 and ** *p* < 0.1 by the Student’s *t*-test. (**C**) Number of *C. muridarum* IFUs recovered from the lungs at day 10 after the i.n. challenge. The median is shown as a horizontal line. Each symbol represents an animal. * *p* < 0.05 by the Mann–Whitney *U* test.

By 10 d.p.c., the mice immunized with *C. muridarum* EBs had only lost −0.3 ± 0.9% of their initial body weight (Figure 7A and Table S2). In contrast, the mice vaccinated with control MOMP or PBS had lost significantly more body weight compared to their initial body weight (−19.7 ± 2.4% and −22.9 ± 1.7%, respectively (*p* < 0.05). The four groups of mice vaccinated with adjuvant combinations had lost less body weight at 10 d.p.c. than the controls receiving MOMP alone or PBS (*p* < 0.05). Significant differences in body weight loss at 10 d.p.c. were found between the groups immunized with IVAX-2 (−8.3% ± 2.3) and CpG-1018+Mont (−2.4% ± 1.2) (*p* > 0.05).

### 3.5. Lung Weights following the i.n. Challenge

As a parameter of local inflammatory responses, which results in the accumulation of cells and fluid in the lungs, lung weights in grams were determined at 10 d.p.c. (Figure 7B and Table S1). The mean lung weight of the mice vaccinated with *C. muridarum* EBs (0.18 ± 0.04) was significantly lower than those of the negative control mice immunized with MOMP alone (0.28 ± 0.01) or PBS (0.32 ± 0.01) (*p* < 0.05). In comparison to the PBS group, the lung weights from the four groups of mice vaccinated with the adjuvant combinations were significantly different (*p* < 0.05). Additionally, the mice vaccinated with IVAX-1 (0.23 ± 0.02), CpG-1018+Mont (0.21 ± 0.01) or CpG-1018+Mont (0.22 ± 0.01) had lighter lungs (*p* < 0.05) than those with MOMP only, while the mice immunized with IVAX-2 (0.26 ± 0.01) did not (*p* > 0.05).

### 3.6. Burden of C. muridarum Infection in the Lungs

To determine the number of *C. muridarum* IFUs in the lungs, ten days after the i.n. challenge, the mice were euthanized and their lungs cultured (Figure 7C and Table S1). The median number of IFUs recovered from the lungs of mice vaccinated with *C. muridarum* EBs was 100 (range < 50–200). In the mice vaccinated with MOMP only or PBS, the median numbers of IFUs recovered were 1,845,250 × 10^3^ and 335,775 × 10^3^, respectively (*p* < 0.05). The mice immunized with MOMP, with the four different combinations of adjuvants, had significantly less IFUs than the mice immunized with MOMP or PBS (*p* < 0.05). Both groups vaccinated with CpG+Montanide had less IFUs than the groups immunized with IVAX formulations (*p* < 0.05). The animals vaccinated with CpG-1018+Mont had the lowest number of IFUs in the lungs (19,360; range 1400–5324 × 10^3^) while the mice immunized with IVAX-2 had the highest number of IFUs (13,976 × 10^3^; range 539 × 10^3^–103,455 × 10^3^; *p* < 0.05).

### 3.7. Local Immune Responses in the Lungs at 10 d.p.c.

**Figure 8:**
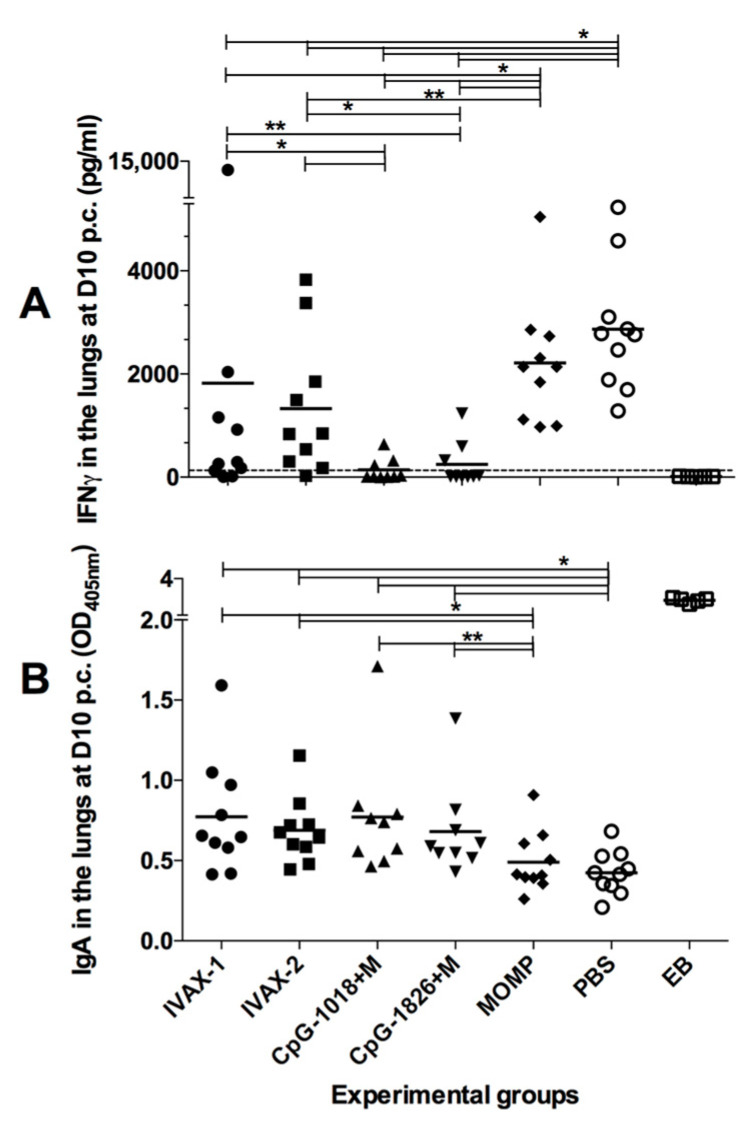
Immune responses in the lungs of mice at 10 d.p.c. (**A**) IFN-γ levels in lung supernatants at 10 d.p.c. The mean is shown as a horizontal line. Each symbol represents an animal. * *p* < 0.05 and ** *p* < 0.1 by the Mann–Whitney *U* test. (**B**) *C. muridarum*-specific IgA levels in lung supernatants at 10 d.p.c. The mean is shown as a horizontal line. Each symbol represents an animal. * *p* < 0.05 and ** *p* < 0.1 by the Student’s *t*-test.

To evaluate local immune responses following the challenge, lung supernatants were collected at 10 d.p.c., and the levels of IFN-γ and *C. muridarum*-specific IgA were determined (Figure 8A,B and Table S1). The mean level of IFN-γ (pg/mL) in the mice vaccinated with *C. muridarum* EBs was below the limit of detection (20 pg/mL), indicating local control of the infection. Levels of IFN-γ were significantly higher in the mice immunized with MOMP only, (2215 ± 382) or PBS (2868 ± 389) (*p* < 0.05), supporting a still active local infection (Figure 8A). The mice immunized with CpG-1018+Mont (247 ± 142) or CpG-1018+Mont (142 ± 75) had significantly lower levels of IFN-γ than the groups immunized using IVAX-1 (1825 ± 1284) or IVAX-2 (1329 ± 420) (*p* < 0.05).

As an indicator of the local humoral immune responses elicited by the vaccination followed by the challenge, the levels of *C. muridarum*-specific IgA (OD_405_) in lung supernatants were determined (Figure 8B and Table S1). The mice vaccinated with *C. muridarum* EBs had the highest median levels of IgA (2.87 ± 0.06), significantly different from those of the animals immunized with MOMP (0.49 ± 0.08) or PBS (0.42 ± 0.04) (*p* < 0.05). The four experimental groups had higher levels of IgA than the controls receiving PBS (*p* < 0.05). IgA levels were not significantly different among the groups of mice vaccinated with the four adjuvant combinations (*p* > 0.05). However, only the mice vaccinated with IVAX-1 (0.77 ± 0.11) or IVAX-2 (0.89 ± 0.08) had higher levels of IgA in comparison with those receiving MOMP only (*p* < 0.05).

## 4. Discussion

The goals of this study were to evaluate in a mouse model the safety and protective efficacy of vaccines formulated with the *C. muridarum* MOMP and four adjuvant combinations. At the site of immunization, the mice immunized using Montanide ISA 720 VG developed a significant inflammatory response, while those receiving IVAX-1 or IVAX-2 did not. The four adjuvant combinations elicited humoral and cellular immune Th1-biased responses. The highest in vitro neutralization titers in serum were induced by IVAX-1, while the highest levels of IFN-γ from T-cell culture supernatants were observed in animals immunized using CpG-1018+Mont. As determined by changes in body weight, weight of the lungs, and number of *C. muridarum* IFUs recovered from the lungs, when compared with the mock-immunized groups receiving PBS or MOMP only, the four formulations were protective. Overall, the CpG-1018+Mont adjuvanted vaccine elicited the most robust protection, while the IVAX-2 formulation induced the lowest levels of protection against the *C. muridarum* respiratory challenge. As expected, the most robust immune responses and best protection was achieved by i.n. immunization with live *C. muridarum* EBs. In our opinion, vaccination with live EBs is not a safe or practical approach to implement in humans. Whether or not it is possible to formulate a subunit vaccine that is as effective as a live vaccine requires further investigation. These results support previous findings indicating that Th1-biased cellular-mediated immune responses producing IFN-γ play a more important role than antibodies in protection against a *C. muridarum* challenge. However, considering the local reactogenicity of adjuvant combinations containing Montanide, the IVAX-1 formulation could be more applicable for the implementation of a chlamydial vaccine in humans.

The benefits and risks of a vaccine need to be carefully evaluated in animal models before it is tested in a Phase I clinical trial [28]. For diseases that have high morbidity and mortality, such as severe viral and bacterial infections and cancer, moderate local and systemic reactogenicity/toxicity may be acceptable since the benefits outweigh the risks. In contrast, in vaccination against diseases with low morbidity and no mortality, such as chlamydial infections, the safety of the vaccination should be a primary consideration. Montanide ISA 720 VG can be prepared as a water-in-oil, water-in-oil-in-water, or as an oil-in-water formulation [37]. In our experience, the water-in-oil suspension, in comparison to the other two formulations, induces the more robust protective immune responses but also the most significant local reactogenicity (unpublished results). The increases in immune responses by the water-in-oil formulation are likely the result of the depot effect that allows the slow release of the antigen and the induction of robust innate responses that trigger adaptive immunity [37].

Protection against a primary *Chlamydia* infection is dependent on a robust cellular immune response [9]. In contrast, for protection against a secondary infection, both antibodies and cell-mediated immune responses play significant roles [9]. Specifically, CD4+ Th1 cells, producing IFN-γ, are the main immune components involved in protection [31,69]. CD8+ T-cells play a secondary role, not due to their cytotoxicity, but likely by secreting IFN-γ [70,71]. Antibodies are a protective factor during the early stages of the infection [69]. Vaccination, therefore, has to elicit both humoral and cellular immune responses to optimize protection against a chlamydial infection.

We have tested several vaccines containing MOMP with a single adjuvant or adjuvant combinations [34,62,72,73,74]. So far, with single adjuvants, we have failed to induce robust protection [72]. However, a combination of a Th1 and a Th2 adjuvants, specifically CpG-1018+Mont, have elicited significant protection in mice against genital and respiratory challenges [17,18,74,75]. To evaluate the safety and efficacy, in addition to CpG-1826, we also tested CpG-1018, an adjuvant recently approved for human use [36]. The local reactogenicity was similar in mice immunized with Montanide ISA 720 VG and CpG-1018 or CpG-1826. ELISA and neutralizing antibody titers were equivalent for both adjuvant combinations. Although the differences were not statistically significant, the levels of IFN-γ in supernatants from T-cells were higher in mice immunized with CpG-1018 versus CpG-1826. Degrees of protection, as evaluated by changes in body weight, weight of the lungs and number of *C. muridarum* IFUs recovered from the lungs, were also similar between the two CpG formulations. Based on these findings, we conclude that CpG-1018, although not significantly superior in this mouse model to CpG-1826, has the advantage that it is already approved for human use and is also effective in mice.

IVAX-1, formulated with influenza A hemagglutinin, has been found to be safe and efficacious when used to elicit neutralizing antibodies in mice [43], and against a respiratory challenge with influenza A virus (manuscript in preparation). Here, we also observed that IVAX-1 and IVAX-2 elicited no significant reactogenicity at the site of immunization. IVAX-1 induced very high levels of neutralizing antibodies when compared with IVAX-2. The quantities of IFN-γ in T-cell supernatants were also higher, but not statistically significant different between the two adjuvants. Similarly, while the changes in body weight, lung weights, and number of *C. muridarum* IFUs in the lungs showed better protection for IVAX-1 versus IVAX-2, the differences were not statistically significant. Neutralizing antibodies likely play a role in protection during the early stages of infection before the EBs are endocytosed by the host cells. Antibodies have been found to play a role also during the intracellular development of *Chlamydia* [76]. Additionally, when a chlamydial inclusion ruptures the host cells, antibodies can block the infection of other host cells. Therefore, although we do not have definitive data to confirm this conclusion to be likely, in the mice immunized with IVAX-1, the high levels of neutralizing antibodies partially blocked the infectivity of *C. muridarum* used to challenge the mice.

A comparison of the results obtained between the IVAX-1 and IVAX-2 versus the two combinations of Montanide ISA 720 VG confirms previous results indicating that cellular immune responses, specifically the secretion by T-cells of IFN-γ, are better indicators of protection than levels of neutralizing antibodies. For example, Pal et al. [77] tested in CD-1 outbreed mice a vaccine formulated with the *C. trachomatis* serovar E MOMP and SPA08, an adjuvant combination containing Alum plus a TLR4 agonist. SPA08 was prepared with buffers containing different amounts of phosphate. The vaccine with the highest phosphate substitution elicited the highest levels of neutralizing antibodies, while the vaccine with the lowest phosphate substitution induced the highest IFN-γ production by T-cells. The most robust protection against a vaginal challenge with *C. trachomatis* serovar E was observed in mice vaccinated with the formulation containing the lowest phosphate substitution. This effect is thought to result from an increase in the phosphorylated adjuvant that allows the formation of a water layer between the components of the vaccine and therefore, a more rapid release of the antigen after immunization [77].

A potential limitation of this study is the use of the respiratory rather than the genital tract model to test the four adjuvant combinations. The respiratory challenge model with *C. muridarum* has extensively been used to evaluate humoral and cellular immune responses to this pathogen and to test vaccines [78,79,80,81,82,83,84,85]. In our experience, if we cannot induce protection in the respiratory model, then that vaccine formulation and delivery system will not protect against a genital tract challenge. An advantage of the respiratory model is that it takes half of the time to complete an experiment versus using the genital tract model. Additionally, the resources needed are significantly reduced. From the results obtained with this experiment, we will consider testing the IVAX-1 vaccine formulation in the genital tract model.

To conclude, the innate responses elicited by adjuvants need to be balanced with reactogenicity/toxicity. Currently, we do not know if these differences between adjuvanticity and reactogenicity are qualitatively or quantitatively different [46,86]. The mechanism of action of adjuvants is poorly understood and therefore, we need to take an empirical approach to moving forward with testing adjuvants. IVAX-1 was recently found to be safe [43]. Here, we confirmed that IVAX-1 formulated with MOMP was also safe. However, the protection against a *C. muridarum* respiratory challenge was not as effective as that obtained when using CpG-1018+Mont or CpG-1826+Mont, thus confirming our hypothesis that the adjuvant combination that elicits the most robust Th1-biased immune responses will be the most protective. This is not surprising since the IVAX adjuvant combinations were formulated with the goal of eliciting protection against influenza A virus, which can be blocked by neutralizing antibodies, while protection against *Chlamydia* is mainly dependent on cell-mediated immune responses [11,13]. It is not possible to predict whether these findings in the mouse model can be translated to humans. Testing IVAX-1 in a non-human primate model could help to decide if this adjuvant, in addition to being safe, can protect humans against a chlamydial challenge. In the meantime, the search for safe and effective adjuvants to formulate a chlamydial vaccine needs to continue.

## Data Availability

Not applicable.

## Supplementary Materials

The following supporting information can be downloaded at: https://www.mdpi.com/article/10.3390/pathogens12070863/s1, Figure S1: Experimental timeline; Table S1: Vaccines formulations, routes of immunization and number of mice; Table S2: Disease burden, yields of *C. muridarum* IFU, and levels of IFN- and *C. muridarum* specific IgA in lung’s supernatants at 10 d.p.c.

## References

[1] CDC (2021). Sexually Transmitted Disease Surveillance 2019. Prevention DoS.

[2] Gottlieb S.L., Johnston C. (2017). Future prospects for new vaccines against sexually transmitted infections. Curr. Opin. Infect. Dis..

[3] Schachter J., Dawson C.R. (1978). Human Chlamydial Infections.

[4] Beem M.O., Saxon E.M. (1977). Respiratory-Tract Colonization and a Distinctive Pneumonia Syndrome in Infants Infected with *Chlamydia trachomatis*. New Engl. J. Med..

[5] Taylor H.R. (2008). Trachoma: A Blinding Scourge from the Bronze Age to the Twenty-First Century.

[6] Nichols R.L., Bell S.D., Murray E.S., Haddad N.A., Bobb A.A. (1966). Studies on trachoma. V. Clinical observations in a field trial of bivalent trachoma vaccine at three dosage levels in Saudi Arabia. Am. J. Trop. Med. Hyg..

[7] Dawson C., Wood T.R., Rose L., Hanna L. (1967). Experimental Inclusion Conjunctivitis in Man. 3. Keratitis and other complications. Arch. Ophthalmol..

[8] Morrison R.P., Belland R.J., Lyng K., Caldwell H.D. (1989). Chlamydial disease pathogenesis. The 57-kD chlamydial hypersensitivity antigen is a stress response protein. J. Exp. Med..

[9] Farris C.M., Morrison R.P. (2011). Vaccination against *Chlamydia* Genital Infection Utilizing the Murine, *C. muridarum* Model. Infect. Immun..

[10] Rockey D.D., Wang J., Lei L., Zhong G. (2009). Chlamydia vaccine candidates and tools for chlamydial antigen discovery. Expert Rev. Vaccines.

[11] De la Maza L.M., Darville T.L., Pal S. (2021). *Chlamydia trachomatis* vaccines for genital infections: Where are we and how far is there to go?. Expert. Rev. Vaccines..

[12] De la Maza L.M., Pal S., Olsen A.W., Follmann F., Tan M., Hegemann J.H., Sutterlin C. (2020). Chlamydia Vaccines.

[13] Phillips S., Quigley B.L., Timms P. (2019). Seventy Years of *Chlamydia* Vaccine Research—Limitations of the Past and Directions for the Future. Front. Microbiol..

[14] Stephens R.S., Wagar E.A., Schoolniki G.K. (1988). High-resolution mapping of serovar-specific and common antigenic determinants of the major outer membrane protein of *Chlamydia trachomatis*. J. Exp. Med..

[15] Zhong G., Brunham R.C., de la Maza L.M., Darville T., Deal C. (2017). National Institute of Allergy and Infectious Diseases workshop report: “*Chlamydia* vaccines: The way forward”. Vaccine.

[16] De la Maza L.M., Zhong G., Brunham R.C. (2017). Update on *Chlamydia trachomatis* Vaccinology. Clin. Vaccine Immunol..

[17] Pal S., Peterson E.M., de la Maza L.M. (2005). Vaccination with the *Chlamydia trachomatis* Major Outer Membrane Protein Can Elicit an Immune Response as Protective as That Resulting from Inoculation with Live Bacteria. Infect. Immun..

[18] Sun G., Pal S., Weiland J., Peterson E.M., de la Maza L.M. (2009). Protection against an intranasal challenge by vaccines formulated with native and recombinant preparations of the *Chlamydia trachomatis* major outer membrane protein. Vaccine.

[19] Kari L., Whitmire W.M., Crane D.D., Reveneau N., Carlson J.H., Goheen M.M., Peterson E.M., Pal S., de la Maza L.M., Caldwell H.D. (2009). *Chlamydia trachomatis* Native Major Outer Membrane Protein Induces Partial Protection in Nonhuman Primates: Implication for a Trachoma Transmission-Blocking Vaccine. J. Immunol..

[20] Teng A., Cruz-Fisher M.I., Cheng C., Pal S., Sun G., Ralli-Jain P., Molina D.M., Felgner P.L., Liang X., de la Maza L.M. (2012). Proteomic identification of immunodominant chlamydial antigens in a mouse model. J. Proteom..

[21] Tifrea D.F., Pal S., de la Maza L.M. (2020). A Recombinant *Chlamydia trachomatis* MOMP Vaccine Elicits Cross-serogroup Protection in Mice Against Vaginal Shedding and Infertility. J. Infect. Dis..

[22] Carmichael J.R., Pal S., Tifrea D., de la Maza L.M. (2011). Induction of protection against vaginal shedding and infertility by a recombinant *Chlamydia* vaccine. Vaccine.

[23] Abraham S., Juel H.B., Bang P., Cheeseman H.M., Dohn R.B., Cole T., Kristiansen M.P., Korsholm K.S., Lewis D., Olsen A.W. (2019). Safety and immunogenicity of the *chlamydia* vaccine candidate CTH522 adjuvanted with CAF01 liposomes or aluminium hydroxide: A first-in-human, randomised, double-blind, placebo-controlled, phase 1 trial. Lancet Infect. Dis..

[24] O’meara C.P., Armitage C.W., Harvie M.C.G., Timms P., Lycke N.Y., Beagley K.W. (2013). Immunization with a MOMP-Based Vaccine Protects Mice against a Pulmonary *Chlamydia* Challenge and Identifies a Disconnection between Infection and Pathology. PLoS ONE.

[25] Ortiz L., Demick K.P., Petersen J.W., Polka M., Rudersdorf R.A., Van Der Pol B., Jones R., Angevine M., Demars R. (1996). Chlamydia trachomatis major outer membrane protein (MOMP) epitopes that activate HLA class II-restricted T cells from infected humans. J. Immunol..

[26] Baehr W., Zhang Y.X., Joseph T., Su H., Nano F.E., Everett K.D., Caldwell H.D. (1988). Mapping antigenic domains expressed by *Chlamydia trachomatis* major outer membrane protein genes. Proc. Natl. Acad. Sci. USA.

[27] Fitch W.M., Peterson E.M., De La Maza L.M. (1993). Phylogenetic analysis of the outer-membrane-protein genes of *Chlamydiae*, and its implication for vaccine development. Mol. Biol. Evol..

[28] Plotkin S.A., Orenstein W.A., Offit P.A. (2018). Plotkin’s Vaccines.

[29] Morrison S.G., Morrison R.P. (2005). A Predominant Role for Antibody in Acquired Immunity to Chlamydial Genital Tract Reinfection. J. Immunol..

[30] Morrison R.P., Feilzer K., Tumas D.B. (1995). Gene knockout mice establish a primary protective role for major histocompatibility complex class II-restricted responses in *Chlamydia trachomatis* genital tract infection. Infect. Immun..

[31] Farris C.M., Morrison S.G., Morrison R.P. (2010). CD4+T Cells and Antibody Are Required for Optimal Major Outer Membrane Protein Vaccine-Induced Immunity to *Chlamydia muridarum* Genital Infection. Infect. Immun..

[32] Wagner H. (2009). The immunogenicity of CpG-antigen conjugates. Adv. Drug Deliv. Rev..

[33] Krieg A.M. (2000). Immune effects and mechanisms of action of CpG motifs. Vaccine.

[34] Pal S., Davis H.L., Peterson E.M., de la Maza L.M. (2002). Immunization with the *Chlamydia trachomatis* Mouse Pneumonitis Major Outer Membrane Protein by Use of CpG Oligodeoxynucleotides as an Adjuvant Induces a Protective Immune Response against an Intranasal Chlamydial Challenge. Infect. Immun..

[35] Tifrea D.F., Pal S., le Bon C., Cocco M.J., Zoonens M., de la Maza L.M. (2020). Improved protection against *Chlamydia muridarum* using the native major outer membrane protein trapped in Resiquimod-carrying amphipols and effects in protection with addition of a Th1 (CpG-1826) and a Th2 (Montanide ISA 720) adjuvant. Vaccine.

[36] Girndt M., Pluer M., Dellanna F., Michelsen A.K., Beige J., Toussaint K., Wehweck H.J., Koch M., Rachti S.H., Faust J. (2022). Immunogenicity and safety of a booster dose of the hepatitis B vaccine HepB-CpG (HEPLISAV-B(R)) compared with HepB-Eng (Engerix-B(R)) and HepB-AS04 (Fendrix(R)) in adults receiving hemodialysis who previously received hepatitis B vaccination and are not seroprotected: Results of a randomized, multicenter phase 3 study. Hum. Vaccines Immunother..

[37] Miles A.P., McClellan H.A., Rausch K.M., Zhu D., Whitmore M.D., Singh S., Martin L.B., Wu Y., Giersing B.K., Stowers A.W. (2005). Montanide ISA 720 vaccines: Quality control of emulsions, stability of formulated antigens, and comparative immunogenicity of vaccine formulations. Vaccine.

[38] Huijbers E.J., Femel J., Andersson K., Bjorkelund H., Hellman L., Olsson A.K. (2012). The non-toxic and biodegradable adjuvant Montanide ISA 720/CpG can replace Freund’s in a cancer vaccine targeting ED-B—A prerequisite for clinical development. Vaccine.

[39] Toledo H., Baly A., Castro O., Resik S., Laferté J., Rolo F., Navea L., Lobaina L., Cruz O., Mıíguez J. (2001). A phase I clinical trial of a multi-epitope polypeptide TAB9 combined with Montanide ISA 720 adjuvant in non-HIV-1 infected human volunteers. Vaccine.

[40] Saul A., Lawrence G., Allworth A., Elliott S., Anderson K., Rzepczyk C., Martin L.B., Taylor D., Eisen D.P., Irving D.O. (2005). A human phase 1 vaccine clinical trial of the *Plasmodium falciparum* malaria vaccine candidate apical membrane antigen 1 in Montanide ISA720 adjuvant. Vaccine.

[41] Feitsma E.A., Janssen Y.F., Boersma H.H., van Sleen Y., van Baarle D., Alleva D.G., Lancaster T.M., Sathiyaseelan T., Murikipudi S., Delpero A.R. (2023). A randomized phase I/II safety and immunogenicity study of the Montanide-adjuvanted SARS-CoV-2 spike protein-RBD-Fc vaccine, AKS-452. Vaccine.

[42] Ross T.M., Gokanapudi N., Ge P., Shi H., Richardson R.A., Pierce S.R., Sanchez P., Ullah S., De Luca E., Sautto G.A. (2022). Kinetic of the Antibody Response Following AddaVax-Adjuvanted Immunization with Recombinant Influenza Antigens. Vaccines.

[43] Hernandez-Davies J.E., Dollinger E.P., Pone E.J., Felgner J., Liang L., Strohmeier S., Jan S., Albin T.J., Jain A., Nakajima R. (2022). Magnitude and breadth of antibody cross-reactivity induced by recombinant influenza hemagglutinin trimer vaccine is enhanced by combination adjuvants. Sci. Rep..

[44] Pouliot K., Buglione-Corbett R., Marty-Roix R., Montminy-Paquette S., West K., Wang S., Lu S., Lien E. (2014). Contribution of TLR4 and MyD88 for adjuvant monophosphoryl lipid A (MPLA) activity in a DNA prime–protein boost HIV-1 vaccine. Vaccine.

[45] Weeratna R.D., Makinen S.R., McCluskie M.J., Davis H.L. (2005). TLR agonists as vaccine adjuvants: Comparison of CpG ODN and Resiquimod (R-848). Vaccine.

[46] Pulendran B., Arunachalam P.S., O’hagan D.T. (2021). Emerging concepts in the science of vaccine adjuvants. Nat. Rev. Drug Discov..

[47] Khurana S., Chearwae W., Castellino F., Manischewitz J., King L.R., Honorkiewicz A., Rock M.T., Edwards K.M., Del Giudice G., Rappuoli R. (2010). Vaccines with MF59 Adjuvant Expand the Antibody Repertoire to Target Protective Sites of Pandemic Avian H5N1 Influenza Virus. Sci. Transl. Med..

[48] Galson J.D., Trück J., Kelly D.F., van der Most R. (2016). Investigating the effect of AS03 adjuvant on the plasma cell repertoire following pH1N1 influenza vaccination. Sci. Rep..

[49] Hammerschlag M.R. (2011). Chlamydial and Gonococcal Infections in Infants and Children. Clin. Infect. Dis..

[50] Komaroff A.L., Aronson M.D., Schachter J. (1981). *Chlamydia trachomatis* infection in adults with community-acquired pneumonia. JAMA.

[51] Tack K.J., Peterson P.K., Rasp F.L., O’Leary M., Hanto D., Simmons R.L., Sabath L.D. (1980). Isolation of Chlamydia Trachomatis from The Lower Respiratory Tract of Adults. Lancet.

[52] Grayston J.T., Kuo C.C., Wang S.P., Altman J. (1986). A New *Chlamydia psittaci* Strain, TWAR, Isolated in Acute Respiratory Tract Infections. New Engl. J. Med..

[53] Murdin A.D., Dunn P., Sodoyer R., Wang J., Caterini J., Brunham R.C., Aujame L., Oomen R. (2000). Use of a Mouse Lung Challenge Model to Identify Antigens Protective against *Chlamydia pneumoniae* Lung Infection. J. Infect. Dis..

[54] Finco O., Bonci A., Agnusdei M., Scarselli M., Petracca R., Norais N., Ferrari G., Garaguso I., Donati M., Sambri V. (2005). Identification of new potential vaccine candidates against *Chlamydia pneumoniae* by multiple screenings. Vaccine.

[55] Chacko A., Delbaz A., Walkden H., Basu S., Armitage C.W., Eindorf T., Trim L.K., Miller E., West N.P., John J.A.S. (2022). *Chlamydia pneumoniae* can infect the central nervous system via the olfactory and trigeminal nerves and contributes to Alzheimer’s disease risk. Sci. Rep..

[56] Jantos C.A., Augustin J., Durchfeld-Meyer B., Baumgärtner W., Schiefer H.G. (1998). Experimental genital tract infection with *Chlamydia psittaci* (GPIC agent) in male rats. Infection.

[57] Knittler M.R., Sachse K. (2015). *Chlamydia psittaci*: Update on an underestimated zoonotic agent. Pathog. Dis..

[58] Liang M., Wen Y., Ran O., Chen L., Wang C., Li L., Xie Y., Zhang Y., Chen C., Wu Y. (2016). Protective immunity induced by recombinant protein CPSIT_p8 of *Chlamydia psittaci*. Appl. Microbiol. Biotechnol..

[59] Caldwell H.D., Kromhout J., Schachter J. (1981). Purification and partial characterization of the major outer membrane protein of *Chlamydia trachomatis*. Infect. Immun..

[60] Pal S., Tifrea D.F., Follmann F., Andersen P., de la Maza L.M. (2017). The cationic liposomal adjuvants CAF01 and CAF09 formulated with the major outer membrane protein elicit robust protection in mice against a *Chlamydia muridarum* respiratory challenge. Vaccine.

[61] Pal S., Fielder T.J., Peterson E.M., De La Maza L.M. (1994). Protection against infertility in a BALB/c mouse salpingitis model by intranasal immunization with the mouse pneumonitis biovar of *Chlamydia trachomatis*. Infect. Immun..

[62] Cheng C., Pal S., Tifrea D., Jia Z., de la Maza L.M. (2014). A vaccine formulated with a combination of TLR-2 and TLR-9 adjuvants and the recombinant major outer membrane protein elicits a robust immune response and significant protection against a *Chlamydia muridarum* challenge. Microbes Infect..

[63] Cheng C., Jain P., Pal S., Tifrea D., Sun G., Teng A.A., Liang X., Felgner P.L., de la Maza L.M. (2014). Assessment of the role in protection and pathogenesis of the *Chlamydia muridarum* V-type ATP synthase subunit A (AtpA) (TC0582). Microbes Infect..

[64] Tifrea D.F., Pal S., Popot J.-L., Cocco M.J., de la Maza L.M. (2014). Increased Immunoaccessibility of MOMP Epitopes in a Vaccine Formulated with Amphipols May Account for the Very Robust Protection Elicited against a Vaginal Challenge with *Chlamydia muridarum*. J. Immunol..

[65] Peterson E.M., Zhong G.M., Carlson E., De La Maza L.M. (1988). Protective role of magnesium in the neutralization by antibodies of *Chlamydia trachomatis* infectivity. Infect. Immun..

[66] Pal S., Bravo J., Peterson E.M., de la Maza L.M. (2008). Protection of Wild-Type and Severe Combined Immunodeficiency Mice against an Intranasal Challenge by Passive Immunization with Monoclonal Antibodies to the *Chlamydia trachomatis* Mouse Pneumonitis Major Outer Membrane Protein. Infect. Immun..

[67] Pal S., Cheng X., Peterson E.M., de la Maza L.M. (1993). Mapping of a surface-exposed B-cell epitope to the variable sequent 3 of the major outer-membrane protein of *Chlamydia trachomatis*. J. Gen. Microbiol..

[68] Tifrea D.F., Sun G., Pal S., Zardeneta G., Cocco M.J., Popot J.-L., de la Maza L.M. (2011). Amphipols stabilize the *Chlamydia* major outer membrane protein and enhance its protective ability as a vaccine. Vaccine.

[69] Naglak E.K., Morrison S.G., Morrison R.P. (2017). Neutrophils Are Central to Antibody-Mediated Protection against Genital Chlamydia. Infect. Immun..

[70] Igietseme J.U., Magee D.M., Williams D.M., Rank R.G. (1994). Role for CD8+ T cells in antichlamydial immunity defined by *Chlamydia*-specific T-lymphocyte clones. Infect. Immun..

[71] Lampe M.F., Wilson C.B., Bevan M.J., Starnbach M.N. (1998). Gamma interferon production by cytotoxic T lymphocytes is required for resolution of *Chlamydia trachomatis* infection. Infect. Immun..

[72] Cheng C., Jain P., Bettahi I., Pal S., Tifrea D., de la Maza L.M. (2011). A TLR2 agonist is a more effective adjuvant for a *Chlamydia* major outer membrane protein vaccine than ligands to other TLR and NOD receptors. Vaccine.

[73] Pal S., Luke C.J., Barbour A.G., Peterson E.M., de la Maza L.M. (2003). Immunization with the *Chlamydia trachomatis* major outer membrane protein, using the outer surface protein A of *Borrelia burgdorferi* as an adjuvant, can induce protection against a chlamydial genital challenge. Vaccine.

[74] Pal S., Cruz-Fisher M.I., Cheng C., Carmichael J.R., Tifrea D.F., Tatarenkova O., de la Maza L.M. (2020). Vaccination with the recombinant major outer membrane protein elicits long-term protection in mice against vaginal shedding and infertility following a *Chlamydia muridarum* genital challenge. NPJ Vaccines.

[75] Pal S., Favaroni A., Tifrea D.F., Hanisch P.T., Luczak S.E., Hegemann J.H., de la Maza L.M. (2017). Comparison of the nine polymorphic membrane proteins of *Chlamydia trachomatis* for their ability to induce protective immune responses in mice against a *C. muridarum* challenge. Vaccine.

[76] Armitage C.W., O’Meara C.P., Harvie M.C.G., Timms P., Blumberg R.S., Beagley K.W. (2014). Divergent outcomes following transcytosis of IgG targeting intracellular and extracellular chlamydial antigens. Immunol. Cell Biol..

[77] Pal S., Ausar S.F., Tifrea D.F., Cheng C., Gallichan S., Sanchez V., de la Maza L.M., Visan L. (2020). Protection of outbred mice against a vaginal challenge by a *Chlamydia trachomatis* serovar E recombinant major outer membrane protein vaccine is dependent on phosphate substitution in the adjuvant. Hum. Vaccines Immunother..

[78] Nogueira C.V., Zhang X., Giovannone N., Sennott E.L., Starnbach M.N. (2015). Protective Immunity against *Chlamydia trachomatis* Can Engage Both CD4+ and CD8+ T Cells and Bridge the Respiratory and Genital Mucosae. J. Immunol..

[79] Starkey M.R., Nguyen D.H., Essilfie A.T., Kim R.Y., Hatchwell L.M., Collison A.M., Yagita H., Foster P.S., Horvat J.C., Mattes J. (2014). Tumor necrosis factor-related apoptosis-inducing ligand translates neonatal respiratory infection into chronic lung disease. Mucosal Immunol..

[80] Zhou X., Chen Q., Moore J., Kolls J.K., Halperin S., Wang J. (2009). Critical role of the interleukin-17/interleukin-17 receptor axis in regulating host susceptibility to respiratory infection with *Chlamydia* species. Infect. Immun..

[81] Tifrea D.F., Pal S., Fairman J., Massari P., de la Maza L.M. (2020). Protection against a chlamydial respiratory challenge by a chimeric vaccine formulated with the *Chlamydia muridarum* major outer membrane protein variable domains using the Neisseria lactamica porin B as a scaffold. NPJ Vaccines.

[82] Cheng C., Cruz-Fisher M.I., Tifrea D., Pal S., Wizel B., de la Maza L.M. (2011). Induction of protection in mice against a respiratory challenge by a vaccine formulated with the *Chlamydia* major outer membrane protein adjuvanted with IC31(R). Vaccine.

[83] Meoni E., Faenzi E., Frigimelica E., Zedda L., Skibinski D., Giovinazzi S., Bonci A., Petracca R., Bartolini E., Galli G. (2009). CT043, a protective antigen that induces a CD4+ Th1 response during *Chlamydia trachomatis* infection in mice and humans. Infect. Immun..

[84] Finco O., Frigimelica E., Buricchi F., Petracca R., Galli G., Faenzi E., Meoni E., Bonci A., Agnusdei M., Nardelli F. (2011). Approach to discover T- and B-cell antigens of intracellular pathogens applied to the design of *Chlamydia trachomatis* vaccines. Proc. Natl. Acad. Sci. USA.

[85] Pal S., Mirzakhanyan Y., Gershon P., Tifrea D.F., de la Maza L.M. (2020). Induction of protection in mice against a respiratory challenge by a vaccine formulated with exosomes isolated from *Chlamydia muridarum* infected cells. NPJ Vaccines.

[86] Didierlaurent A.M., Collignon C., Bourguignon P., Wouters S., Fierens K., Fochesato M., Dendouga N., Langlet C., Malissen B., Lambrecht B.N. (2014). Enhancement of Adaptive Immunity by the Human Vaccine Adjuvant AS01 Depends on Activated Dendritic Cells. J. Immunol..

