# Supplementary material for: Evaluation of Four Adjuvant Combinations, IVAX-1, IVAX-2, CpG-1826+Montanide ISA 720 VG and CpG-1018+Montanide ISA 720 VG, for Safety and for Their Ability to Elicit Protective Immune Responses in Mice against a Respiratory Challenge with Chlamydia muridarum
Source: Pathogens. 2023 Jun 22;12(7):863. doi: 10.3390/pathogens12070863 (PMC10383793; doi:10.3390/pathogens12070863)
Supplement: Supplementary file 1 [file pathogens-12-00863-s001.zip › pathogens-2360550-supplementary.pdf]

Figure S1. Experimental timeline.

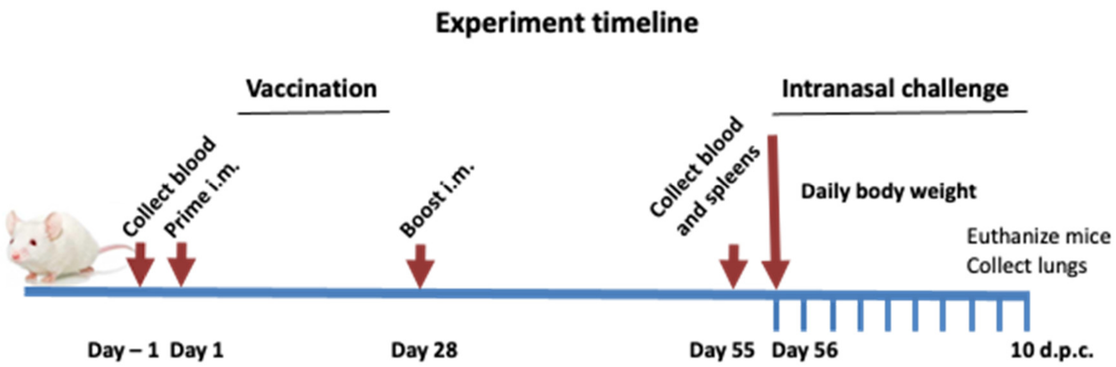

Table S1. Vaccines formulations, routes of immunization and number of mice.

Antigen (µg/dose)	Adjuvants	Routes of Immunization	# of Mice for T-Cell Responses	# of Mice Challenged
rMOMP (10µg)	IVAX-1 (MPLA+CpG1018+AddaVax)	i.m.	3	10
rMOMP (10µg)	IVAX-2 (MPLA+CpG1018+ASO3)	i.m.	3	10
rMOMP (10µg)	CpG-1018+Montanide ISA 720 VG	i.m.	3	9
rMOMP (10µg)	CpG-1826+Montanide ISA 720 VG	i.m.	3	9
rMOMP (10µg)	No adjuvants	i.m.	3	10
Viable EB	No adjuvants	i.n.	3	10
PBS	No adjuvants	i.m.	3	10

Table S2. Disease burden, yields of *C. muridarum* IFU, and levels of IFN-◎ and *C. muridarum* specific IgA in lung's supernatants at 10 d.p.c.

Vaccine	% Change Body Weight (mean ± 1 SE)	Lungs Weight (g) (Mean ± 1SE)	Median Number IFU Recovered from Lungs (min–max) x10^3	IFN-⚜ (pg/ml) (Mean ± 1 SE)	IgA (OD_{405}) (Mean ± 1SE)
MOMP+IVAX-1	−4.9±1.7[a,b]	0.23±0.02[a,b]	2,844 (64–107,690)[f,g]	1,825±1284[a]	0.77±0.11[a,b]
MOMP+IVAX-2	−8.3±2.3[a,b,c]	0.26±0.01[a]	13,976 (539–103,455)[f,g,h]	1,329±420[a]	0.89±0.08[a,b]
MOMP+CpG-1018+M	−2.4±1.2[a,b]	0.22±0.01[a,b,d]	19 (1.4–5,324)[f,g,h,i]	142±75[a,b]	0.77±0.13[a,e]
MOMP+CpG-1826+M	−3.3±1.0[a,b]	0.21±0.01[a,b,d]	212 (0.15–41,140)[f,g,h,i]	247±142[a,b]	0.68±0.10[a,e]
MOMP (− control)	−19.7±2.4	0.28±0.01	1,845,250 (187,550–4,053,500)	2,215±382	0.49±0.08
PBS (− control)	−22.9±1.7	0.32±0.01	335,775 (108,900–5,082,000)	2,868±389	0.42±0.04
EB (+ control)	−0.3±0.9	0.18±0.004	0.1 (< 0.005–0.2)	3±3	2.87±0.06

[a] — $p < 0.05$ by the Student's *t* test compared to the PBS immunized mice. [b] — $p < 0.05$ by the Student's *t* test compared to the MOMP only immunized mice. [c] — $p < 0.05$ by the Student's *t* test compared to the IVAX-2 immunized mice. [d] — $p = 0.5$ by the Student's *t* test compared to the IVAX-2 immunized mice. [e] — $p < 0.05$ by the Mann-Whitney U test compared to the MOMP only immunized mice. [f] — $p < 0.05$ by the Mann-Whitney U test compared to the PBS immunized mice. [g] — $p < 0.05$ by the Mann-Whitney U test compared to the MOMP only immunized mice. [h] — $p < 0.05$ by the Mann-Whitney U test compared to the IVAX-1 immunized mice. [i] — $p < 0.05$ by the Mann-Whitney U test compared to the IVAX-2 immunized mice.
